# A Facile Strategy for Immobilizing GOD and HRP onto Pollen Grain and Its Application to Visual Detection of Glucose

**DOI:** 10.3390/ijms21249529

**Published:** 2020-12-15

**Authors:** Shanxia Jin, Liping Liu, Mengying Fan, Yaru Jia, Ping Zhou

**Affiliations:** 1 School of Chemistry and Environmental Engineering, Wuhan Institute of Technology, Wuhan 430205, China; lock1_8@whu.edu.cn; 2 College of Chemistry and Molecular Sciences, Wuhan University, Wuhan 430072, China; lpliu@whu.edu.cn (L.L.); myfan@whu.edu.cn (M.F.); 2017202030052@whu.edu.cn (Y.J.)

**Keywords:** enzyme immobilization, boronate affinity, pollen grain, glucose detection

## Abstract

Pollen grain was explored as a new carrier for enzyme immobilization. After being modified with boric acid-functionalized titania, the pollen grain was able to covalently immobilize glycosylated enzymes by boronate affinity interaction under very mild experimental conditions (e.g., pH 7.0, ambient temperature and free of organic solvent). The glucose oxidase and horse radish peroxidase-immobilized pollen grain became a bienzyme system. The pollen grain also worked as an indicator of the cascade reaction by changing its color. A rapid, simple and cost-effective approach for the visual detection of glucose was then developed. When the glucose concentration exceeded 0.5 mM, the color change was observable by the naked eye. The assay of glucose in body fluid samples exhibited its great potential for practical application.

## 1. Introduction

Enzymes are highly efficient biocatalysts that catalyze chemical reactions with great chemo-, regio- and stereo-selectivity under mild reaction conditions, such as ambient temperature, physiological pH, and aqueous environment, etc. [1,2,3]. Enzymes are becoming increasingly important for diverse applications including biocatalysis [4], biomedicine [5], organic synthesis [6], biosensors development [7], environmental protection [8], andfood industry [9]. However, some drawbacks such as the lack of long-term stability, marginal lifetime, and the difficulty in recovery often hamper the use of enzymes in free forms. These drawbacks can often be overcome by immobilizing the enzyme on different solid supports [10]. The use of an enzyme in an immobilized form provides for advantages such as convenience in the handling, ease of separation from the products, recovery and reuse of costly enzymes, and a possible increase in stability under both storage and operational conditions [11].

Some classic methods of enzyme immobilization can be distinguished, including physical adsorption, covalent immobilization, encapsulation, entrapment and cross-linking. Although each strategy has its characteristic pros and cons, mild process conditions are necessary to retain the catalytic properties of immobilized enzymes, and a cost-effective immobilization method is desirable for the practical applications of the immobilized enzyme systems [12]. In comparison with the others, covalent immobilization has many particular merits including strong linkage, little leakage, and multiple groups for availability [13]. However, the chemical modification probably results in the loss of the functional conformation of enzymes. Sometimes the essential linkers such as glyoxal, epichlorohydrin or glutaraldehyde have to be added for modification [14]. The application of these additions may request complicated preparations and cause indiscernible problems for the environment and human health.

The boronate affinity relies on the specific esterification reaction of boronic acid ligands with the 1,2 or 1,3 cis-diol moieties of target molecules and has gained increasing interest for its selective enrichment of cis-diol-containing biomolecules [15,16]. The glycosylated enzymes, which account for a large proportion of enzymes, can be covalently bound by boronate affinity interaction [17,18]. It does not only provide a stable, specific, and reusable technique for enzyme immobilization, but also can excellently maintain the structural and functional properties of enzymes because boronic acids react with 1,2-diols in the glycosylated regions where normally hardly affected the catalytic activities of enzymes [19,20]. However, almost all ligands used in boronate affinity materials are aromatic boronic acids, which may be a source of nonspecific hydrophobic interaction [21]. While hydrophobic interaction can be reduced by the addition of some percentage of organic solvents [22], the risk of the denaturation of enzymes may arise from the organic solvents. Recently, some new materials with improved hydrophilicity by using inorganic boric acid as affinity ligands have emerged. They showed good potential for glycoprotein enrichment [23,24,25].

The selection of the appropriate support matrix is important for optimal immobilization to preserve the activity and stability of the enzyme. Naturally derived biopolymers as support have attracted significant attention over the past several years owing to their abundance and versatile properties such as non-toxicity, biocompatibility, biodegradability and renewability [26,27]. In this work, pine pollen grains were explored as a support because they have regular shape, uniform particle-size, endogenous hydrophilicity, and numerous reactive sites for incorporating novel functionalities. The pollen grains were modified with inorganic boric acid-functionalized titania, and then glucose oxidase (GOD) and horse radish peroxidase (HRP) were immobilized onto pollen grains by boronate affinity interaction under very mild experimental conditions. On the basis of the specific properties of the modified pollen grains, a simple method for visual determination of glucose was developed and was employed to recognize glucose in urine and blood samples.

## 2. Results and Discussion

### 2.1. Characterization of Modified Pollen Grains

The modified pollen grains were prepared via hydrolysis of a titanium precursor, tetrabutyl orthotitanate, followed by condensation in a suspension containing pollen grains and boric acid. Boric acid was incorporated into titania in the process of polycondensation. Similarly, the boric acid-functionalized titania was attached to pollen grains through the hydroxyl functional groups on the surface of pollen grains during the sol–gel process.

**Figure 1:**
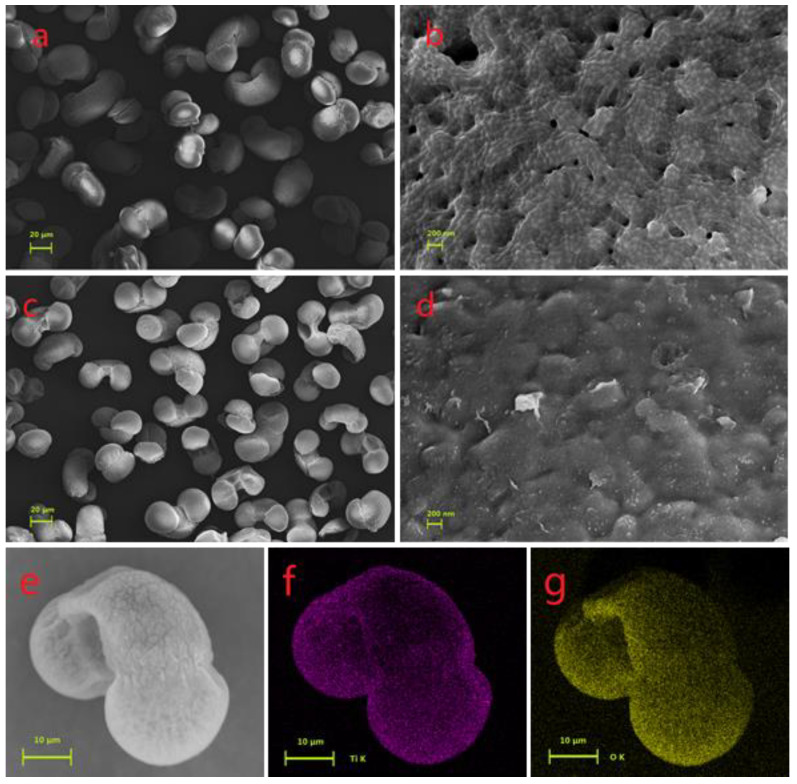
SEM photographs of the pollen grains before (**a**,**b**) and after (**c**–**e**) being modified with boric acid-functionalized titania, and the energy dispersive X-ray mapping images of the modified pollen grain for the identification of titanium (**f**) and oxygen (**g**). (**a**,**c**) 200×; (**b**,**d**) 20000×; (**e**–**g**) 1700×.

The morphologies of the pollen grains before and after being modified with boric acid-functionalized titania were characterized by scanning electron microscope (SEM). As shown in Figure 1a, unmodified pollen grains were dumbbell-like with an average size of long shaft about 60–70 μm and a short shaft of about 30–40 μm. The photograph at high magnification revealed that the surface of the pollen grain had a fiber-like architecture with some nanopores (Figure 1b). After modification (Figure 1c,d), the surface was covered by boric acid-functionalized titania. When a modified pollen grain (Figure 1e) was analyzed by the energy-dispersive X-ray (EDX) spectroscopy, it revealed the existence of titanium (Supplementary Figure S1). The EDX mapping images of the single modified pollen grain for Ti and O elements are shown in Figure 1f,g, respectively. Titania was demonstrated to be evenly distributed over the pollen grain. Supplementary Figure S2a shows the X-ray photoelectron spectroscopy (XPS) survey spectrum of the modified pollen grains. The peaks indicated that the surface elemental composition was made of C, O, Ti, N and B (Supplementary Table S1). The high-resolution XPS spectrum of the B 1s region contained a peak at 191.78 eV (Supplementary Figure S2b), which was attributable to the formation of bonds between boric acid and titania [28].

### 2.2. Enzyme-Immobilization on the Pollen Grains 

**Scheme 1:**
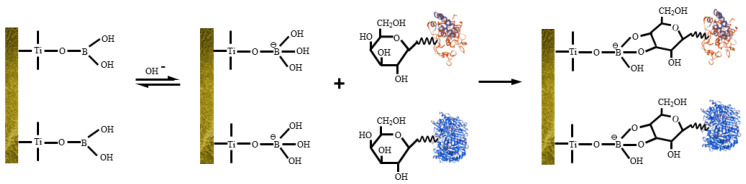
The interaction between enzymes and the modified pollen grain.

In the aqueous environment, 1,2- or 1,3- diol moieties of the enzymes reacted with boric acid ligands on the surface of the modified pollen grain creating cyclic ester (Scheme 1). The interaction mechanism is similar to the reversible esterification between organic boronic acids and cis-diol-containing compounds.

**Figure 2:**
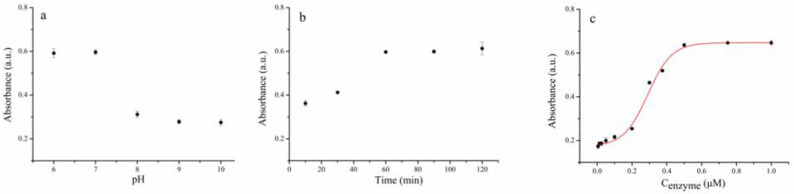
Effects of (**a**) loading pH, (**b**) loading time, and (**c**) initial concentration of enzymes during immobilization. Data were obtained from the average values of three replicated measurements.

The pH environment does not only influence the activities of enzymes but is also a crucial factor for boronate affinity. Firstly, the optimal pH for enzyme immobilization was assessed. As shown in Figure 2a, the highest absorbance value was observed at pH 7.0. HRP is suitable to be used in the pH range from 3.0 to 9.0, and GOD favors being used between pH 4.0 and 7.0 [29]. To prevent denaturation, a neutral condition was chosen for enzyme immobilization, although the affinity of boric acid towards cis-diol-containing compounds prefers higher pH conditions. The effects of loading time and initial concentration of enzymes on immobilization were then investigated. As shown in Figure 2b, the signal increased with the increase in loading time, and kept nearly steady from 60 to 120 min. When the initial enzyme concentrations were above 0.7 μM, the signal was kept relatively constant (Figure 2c). Thus, for the enzyme immobilization, the appropriate loading time was set as 60 min and the initial enzyme concentrations were chosen as 0.7 μM. Under optimized immobilization conditions, the amount of immobilized GOD or HRP on pollen grains (pH 7.0) were tested (Supplementary Table S2). When two enzymes were immobilized simultaneously, the amounts of GOD and HRP were measured to be about 14.20 and 5.11 μg/mg pollen grains, respectively, slightly lower than those of individual immobilized enzymes. The immobilization efficiencies of GOD and HRP were measured to be 32 and 15%, respectively.

The kinetic properties of free and immobilized enzymes were summarized in Table 1. The Michaelis constantKm $K_{m}^{app}$ and the catalytic constant $k_{cat}$ were obtained from the Lineweaver–Burk plots (Supplementary Figure S3). The results showed that $K_{m}^{app}$ value of free HRP was lower than that of immobilized HRP, indicating a decreased enzyme–substrate affinity for the immobilized enzyme. The difference for GOD is minor.

### 2.3. The Visual Detection of Glucose

**Scheme 2:**
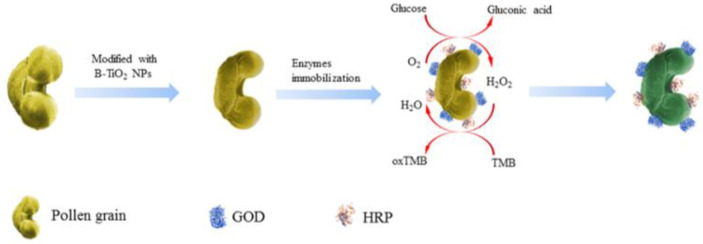
Proposed mechanism of the enzyme immobilization onto the pollen grain and the visual determination of glucose.

The preliminary experiments for the application of enzyme-immobilized pollen grains were performed in microcentrifuge tubes. When the pollen grains were added to the solutions containing 3,3′,5,5′-tetramethyl benzidine (TMB) and different monosaccharides, the color of the suspensions containing glucose changed as expected. Furthermore, the pollen grain significantly changed its color. Supplementary Figure S4 showed the color change of the remaining pollen grains after removing the reaction solutions by centrifugation. When glucose solution was incubated with unimmobilized pollen grains or only HRP-immobilized pollen grains, the color remained unchanged (Supplementary Figure S5). The protocol for enzyme immobilization onto pollen grains and the mechanism for glucose detection are illustrated in Scheme 2. The modified pollen grain worked as a carrier for GOD and HRP, and a cascade enzymatic reaction occurred when both glucose and TMB were present. GOD used glucose as a substrate to produce gluconolactone and hydrogen peroxide. As a substrate of HRP, TMB was oxidized by the HRP/H_2_O_2_ system to yield colored products [30,31]. The adsorption of oxidation products of TMB onto the pollen grain resulted in the color change.

**Figure 3:**
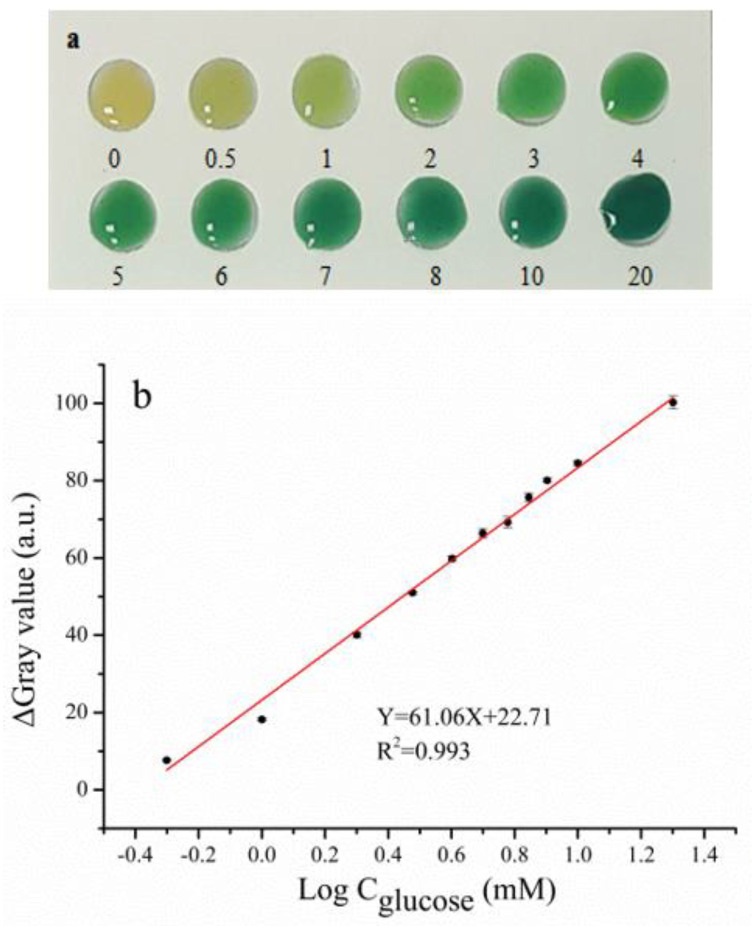
Digital image of a test slide (**a**) and the linearity of relative color intensity with respect to glucose at different concentrations (**b**). The concentration of glucose in 10 mM NH_4_Cl buffer solution (pH 7.4) was 0 to 20 mM, respectively. The error bars represent the mean ±SD of five independent experiments.

Instead of microcentrifuge tubes, a glass slide was used for the convenience of quantification analysis. The performance of the enzyme-immobilized pollen grains for the detection of glucose standards in buffer solution was evaluated. As shown in Figure 3a, the color of the detection zones on the slide changed from yellow to green when the concentrations of glucose increased. The color change was observable by the naked eye in the presence of 0.5 mM glucose. When the image was processed by ImageJ software, it showed that the ΔGray values increased linearly with the logarithms of the concentrations of glucose within the linear range from 0.5 to 20 mM (Figure 3b). The relative standard deviations (RSD) were less than 10% (*n* = 5), and the limit of detection (LOD, which was calculated from the regression equation 3S_0_/S, where S_0_ was the standard deviation of blank samples (*n* = 10), and S was the slope of the calibration curve) was 0.16 mM (Supplementary Table S3). The sensitivity was inferior to those of visual methods based on fluorescent detection [32,33,34], but comparable to those of some visual methods based on colorimetric detection [30,35,36]. These results demonstrated that the enzyme-immobilized pollen grains could be served as a sufficient and efficacious indicator for the rapid and sensitive detection of glucose via vivid color change.

### 2.4. Selectivity of Glucose Detection 

**Figure 4:**
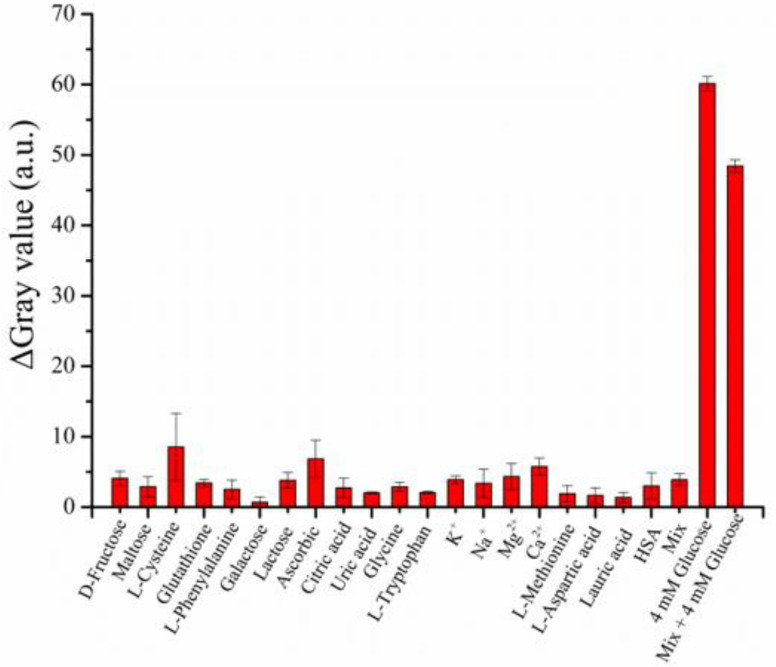
Selectivity of the enzyme-immobilized pollen grains towards various potential interfering substances. All interfering substances were prepared in 10 mM NH_4_Cl buffer (pH 7.4) at 10-fold concentrations of those listed in Table S4. The mix contained all of the interfering substances.

Owing to the complexities of the body fluid samples, the proposed analytical methods should be with high selectivity in practical applications. To investigate the selectivity of this pollen grains-based method, the control solutions containing different substances which were the main potential interferences with competitive effects to glucose in blood or urine were tested [37]. All the substances were prepared at 10-fold concentrations of their normal levels in human body fluids (listed in Supplementary Table S4). As shown in Figure 4, the ΔGray values obtained with glucose were much higher than the controls and remained greater than 80% with the mixture of all agents. The results demonstrated that these substances had little impact on the enzyme-immobilized pollen grains, and the established method had potential for the determination of glucose in body fluids.

### 2.5. Analysis of Glucose in Human Urine and Blood

Encouraged by the aforementioned experimental results, we used this method to evaluate the glucose levels in human urine samples. The glucose concentrations in healthy urine samples were normally lower than 1 mM [37], so glucose was spiked into the urine sample of a healthy volunteer. The response of test slide for the spiked samples with different concentrations of glucose was shown in Supplementary Figure S6a. The colors of the detection zones varied obviously with the change of glucose concentration from 0 to 20 mM. Six urine samples from four healthy volunteers and two diabetic patients were tested by this array (Supplementary Figure S6b). By comparing with the colors of spiked samples in Supplementary Figure S6a, the glucose levels in the urine samples (No.I–No.IV) from healthy volunteers were estimated to be lower than 0.5 mM. The No.V and No.VI samples were positive since the concentrations of glucose were obviously higher than 2.8 mM [38]. The ΔGray value of sample No.V exceeded that of the spiked sample at the highest concentration. The quantitative result of sample No.VI was in good agreement with that measured by ion chromatography (Table 2). The results of recovery experiments also indicated the method had high accuracy in the analysis of urine samples (Supplementary Table S3). This assay does not only provide a quick and visible way to distinguish the glucose levels corresponding to either healthy or diabetic stages, but also allows the quantitative diagnoses of diabetes patients from the concentrations of glucose in urine.

Normally, the concentration of glucose in the human bloodstream is in the range of 3.8–6.9 mM, and the blood glucose level should be controlled below 10 mM for diabetics [39]. To further assess the applicability of the proposed method, plasma samples collected from five healthy volunteers were tested, and the glucose levels were determined by external standard method (Supplementary Figure S7). As shown in Table 3, the results were close to those tested by the glucometer (Roche, Accu-Chek Active).

## 3. Materials and Methods

### 3.1. Materials and Reagents

Tetrabutyl orthotitanate (TBOT), boric acid, ammonium chloride, disodium phosphate, sodium dihydrogen phosphate, methanol, ethanol, acetonitrile, 3,3′,5,5′-tetramethyl benzidine, glucose, fructose, maltose, galactose, lactose, citric acid were obtained from Sinopharm Chemical Reagent Co., Ltd. (Shanghai, China). Uric acid, lauric acid, ascorbic acid, L-aspartic acid were purchased from Aladdin Chemical Reagent Co., Ltd. (Shanghai, China). Bovine serum albumin (BSA), human serum albumin (HSA), horse radish peroxidase, glucose oxidase, L-cysteine, glutathione, L-phenylalanine, glycine, L-tryptophan, L-methionine were purchased from Sigma-Aldrich (St. Louis, MO, USA). Human serum was purchased from Solarbio (Beijing, China).

### 3.2. Instrumentation

The morphology of the prepared material was observed by a Sigma field emission scanning electron microscope (Zeiss, Oberkochen, Germany). The elemental species energy-dispersive X-ray spectroscopy analysis was examined by a field emission electron probe microanalyzer (JXA-8530F Plus, JEOL, Tokushima, Tokyo, Japan). X-ray photoelectron spectroscopy measurement was taken by an ESCALAB 250 Xi electron spectrometer (Thermo Scientific, Waltham, MA, USA) using a radiation source of Al Kα radiation with the energy of 1486.6 eV. An ICS 2500 chromatography system (Dionex, CA, USA) with Dionex CarboPac PA 20 (3 × 150 mm) separation column and PA 20 guard (3 × 30 mm) column was used in this study. The mobile phase was 12 mM potassium hydroxide and the flow rate was 0.4 mL/min. The detection was carried out by an integrated pulsed amperometry cell equipped with a working gold electrode and a combined pH–Ag/AgCl reference electrode.

### 3.3. Preparation of Pollen Grains Modified with Boric Acid-Functionalized Titania

The dry pollen grains were immersed in methanol and refluxed at 60 °C for 12 h. The pollen grains were then washed successively by methanol, 1 M HCl, 1 M NaOH and deionized water, and followed by lyophilization. Four hundred milligrams of pollen grains, 30 mL of boric acid solution (48.5 mM in ethanol), 150 μL of saturated boric acid aqueous solution, and 30 mL of boric acid solution (16.2 mM in acetonitrile) were successively added into a flask under constant stirring at room temperature. Two milliliters of TBOT solution (29.3 mM in ethanol) was then added rapidly into the flask and stirred for 8 h at room temperature. The products were washed sufficiently by ethanol and deionized water, and then freeze-dried.

### 3.4. Enzyme Immobilization

The effects of pH, loading time and enzyme concentrations on immobilization were explored to obtain optimal experimental conditions. We added 0.48 mL of buffer solution (10 mM NH_4_Cl) with different pH values to the centrifuge tubes. Then, 1 mg of modified pollen grains, 10 μL of GOD solution (25 μM in deionized water) and 10 μL of HRP solution (25 μM in deionized water) were added to each tube. After incubation at 25 °C for 2 h, the mixtures were centrifuged at 6000 rpm for 5 min. The supernatants were discarded and the pollen grains were washed five times by deionized water. Then, 300 μL of NH_4_Cl buffer solution (10 mM, pH 7.0), 150 μL of TMB solution (25 mM in deionized water), and 50 μL of glucose solution (50 mM in deionized water) were subsequently added to each tube. After incubation for 6 min, 150 μL of 2 M H_2_SO_4_ was added. After centrifugation at 10,000 rpm for 5 min, the supernatants were analyzed by a multifunctional microplate reader (Tecan, Salzburg, Austria), and the absorption values at 450 nm were recorded. To assess the effects of loading time, the loading pH was set at 7.0 and the loading time was varied from 10 to 120 min, and the other experimental procedures were the same as above. For the study of initial enzyme concentrations on immobilization, the loading pH was set at 7.0 and the loading time was 120 min. The molar ratio of GOD and HRP was kept as 1:1, and the concentration of each enzyme was varied from 0.005 to 1 μM. The other experimental procedures were kept unchanged.

To determine the amounts of adsorption, 0.5 mL of GOD or/and HRP solution (0.7 μM each in 10 mM NH_4_Cl buffer, pH 7.0) and 1 mg of modified pollen grains were added to a centrifuge tube, and the mixture was then incubated at 25 °C for 1 h. The adsorption amounts of GOD and HRP on pollen grains were calculated by subtracting the unbound enzymes from the total amount of enzymes monitored by UV–vis spectroscopy.

To assess the efficiency of immobilization, the catalytic activities of free or immobilized enzymes were determined by following the rate of production formation in an HRP/TMB assay reaction. In addition to 0.5 mL of GOD and HRP solution (0.7 μM each in 10 mM NH_4_Cl buffer, pH 7.0), 1 mg of modified pollen grains were added to a centrifuge tube. The mixture was incubated at 25 °C for 1 h. The assay solution for HRP is a mixture of 2.5 mM H_2_O_2_ and TMB each in 10 mM pH 7.0 NH_4_Cl buffer. The assay solution for GOD was a mixture of 50 mM glucose, 2.5 mM TMB and 0.037 μM HRP in 10 mM pH 7.0 NH_4_Cl buffer. The reaction was monitored in real time via the increase in the TMB oxidation product absorption at 652 nm. The same experiment was repeated for the initial free enzyme, the free enzyme in the supernatant and the enzyme immobilized on the pollen grains. The activity was calculated via the initial rate method by utilizing the initial linear portion of the 652 nm absorbance time course. The efficiency of immobilization was calculated by dividing the activity of the immobilized enzyme with the activity difference between initial free enzyme and free enzyme in the supernatant.

### 3.5. Kinetic Assays of Free and Immobilized Enzymes

The catalytic kinetic assays were carried out at room temperature by using UV–vis spectra. NH_4_Cl buffer solution (10 mM, pH 7.0) was used for the preparation of substrate or enzyme solutions. For the kinetic assay of TMB, 100 μL of glucose (10 mM) and 10 μL of a mixture of GOD and HRP (14.20 μg/mL and 5.11 μg/mL, respectively) or a suspension of enzyme-immobilized pollen grains (1 mg/mL) was added to 100 μL of TMB solution at varied concentrations. For the kinetic assay of glucose, 100 μL of TMB (0.5 mM) and 10 μL of a mixture of GOD and HRP (14.20 μg/mL and 5.11 μg/mL, respectively) or a suspension of enzyme-immobilized pollen grains was added to 100 μL of glucose solution at varied concentrations. The catalytic reaction was allowed to proceed for 4 min, and then stopped by adding 200 μL of 2 M H_2_SO_4_. The rates of the reactions were evaluated from the initial slopes of the time-dependent absorbance curves at 450 nm.

### 3.6. Preparation of Samples

The urine samples of healthy volunteers or diabetic patients were centrifuged at 8000 rpm for 15 min, and the supernatants were collected. The whole blood samples from healthy volunteers were centrifuged at 6000 rpm for 15 min to obtain the plasma samples. The human serum sample was diluted with 10 mM NH_4_Cl buffer (pH 7.4). For determining the calibration curve, glucose solutions at different concentrations were made by spiking glucose into the urine sample or 10-fold diluted serum.

### 3.7. Procedure for the Detection of Glucose 

Ten milligrams of enzyme-immobilized pollen grains were dispersed in 1 mL of 10 mM NH_4_Cl buffer (pH 7.4). Three microliters of pollen grain suspension, 3 µL of TMB solution (7.2 mM in deionized water), and 3 µL of sample or glucose standard at concentration ranging from 0.5 to 20 mM in 10 mM NH_4_Cl buffer (pH 7.4) were successively added to the circle zones of the glass slide. Then the mixtures were incubated at 25 °C for 6 min, and the images were recorded by a smartphone (Meizu pro 6). For each detection zone, a constant region of interest was selected to calculate the gray value by ImageJ software. The corrected gray value (shorted as ΔGray value) was the gray value of the enzyme-immobilized pollen grains acquired before the sample application subtracted the gray value acquired after the sample application.

## 4. Conclusions

In summary, glycosylated enzymes were successfully immobilized onto pine pollen grains. As far as we know, this is the first time that pollen grain was used as a solid support for enzyme immobilization. GOD and HRP, as model enzymes, were immobilized onto the modified pollen grains by boronate affinity interaction between inorganic boric acid ligands and the glycosylated domains of enzymes rather than the catalytic domains. The enzymes were covalently immobilized under very mild experimental conditions without using organic solvents and any linkers, and the immobilization procedure was facile and time saving. Owing to the specific properties of enzyme-immobilized pollen grains, a simple approach for the visual determination of glucose has been developed. The pollen grain did not only work as a carrier of enzymes but also as an indicator of the cascade reaction. The new approach is rapid, reliable and cost-effective, and has exhibited great potential for practical applications. This allows the analysis of glucose without the need of special instrumentation. We believe that the modified pollen grain can be evolved into a generic carrier to immobilize glycosylated enzymes and the HRP-immobilized pollen grain can be used for the visual determination of many other analytes.

## Figures and Tables

**Table 1:** Catalytic parameters of the free and immobilized enzymes.

Substrate	Enzyme Status	$K_{m}^{app}\;(\mathrm{mM})$	$k_{cat}\;(s^{-1})$
TMB	Co-immobilized	2.39 × 10^−^^1^	27.58
	Free	5.93 × 10^−2^	24.12
Glucose	Co-immobilized	23.91	31.76
	Free	24.84	40.96

**Table 2:** Detection of glucose in human urine samples.

Sample Number	Proposed Method (mM)	IC (mM)
I	−	0.13
II	−	0.12
III	−	0.12
IV	−	0.15
V	+++	26.12
VI	3.42 ± 0.19	3.53

(“−” and “+” indicate those who are negative or positive to diabetic, *n* = 3).

**Table 3:** Analysis of glucose in human plasma (*n* = 3).

Sample Number	Proposed Method (mM)	Glucometer (mM)
I	7.92 ± 0.22	8.2
II	6.92 ± 0.24	7.4
III	5.14 ± 0.11	4.9
IV	8.13 ± 0.20	8.3
V	5.56 ± 0.44	6.2

## Supplementary Materials

The following are available online at https://www.mdpi.com/1422-0067/21/24/9529/s1.

## Abbreviations

GOD	Glucose oxidase
HRP	Horse radish peroxidase
TMB	3,3′,5,5′-Tetramethyl benzidine
TBOT	Tetrabutyl orthotitanate
SEM	Scanning electron microscope
EDX	Energy-dispersive X-ray
XPS	X-ray photoelectron spectroscopy
