# Supplementary material for: A Facile Strategy for Immobilizing GOD and HRP onto Pollen Grain and Its Application to Visual Detection of Glucose
Source: Int J Mol Sci. 2020 Dec 15;21(24):9529. doi: 10.3390/ijms21249529 (PMC7765182; doi:10.3390/ijms21249529)
Supplement: Supplementary file 1 [file ijms-21-09529-s001.pdf]

Supporting Information

for

A facile strategy for immobilizing GOD and HRP onto pollen grain and its application to visual detection of glucose

Shanxia Jin [1,2], Liping Liu [2], Mengying Fan [2], Yaru Jia [2] and Ping Zhou [2,*]

[1] School of Chemistry and Environmental Engineering, Wuhan Institute of Technology, Wuhan 430205, People's Republic of China
[2] College of Chemistry and Molecular Sciences, Wuhan University, Wuhan 430072, People's Republic of China

* Correspondence: zbping@whu.edu.cn

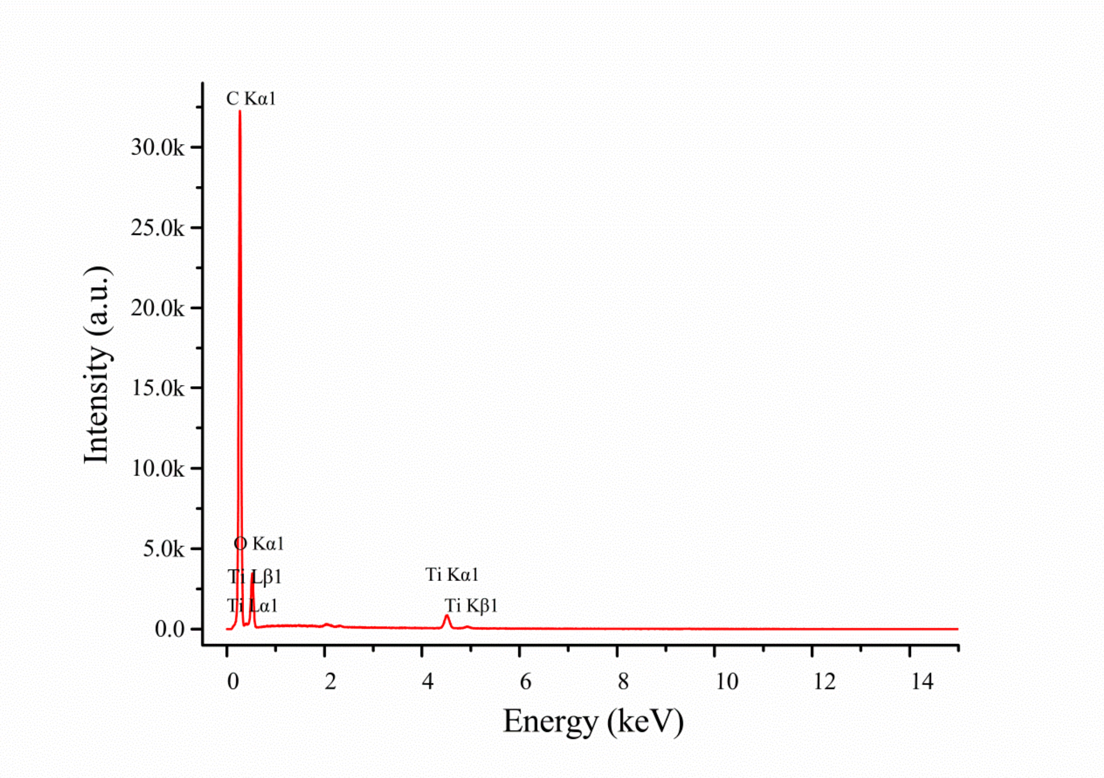

Figure S1. EDX spectrum of a modified pollen grain.

Table S1 The atom concentration ratios of modified pollen grains.

Name	O 1s	C 1s	Ti 2p	B 1s	N 1s
Peak BE	530.15	284.78	458.54	191.78	399.93
Atomic %	34.47	46.45	15.36	1.59	2.12

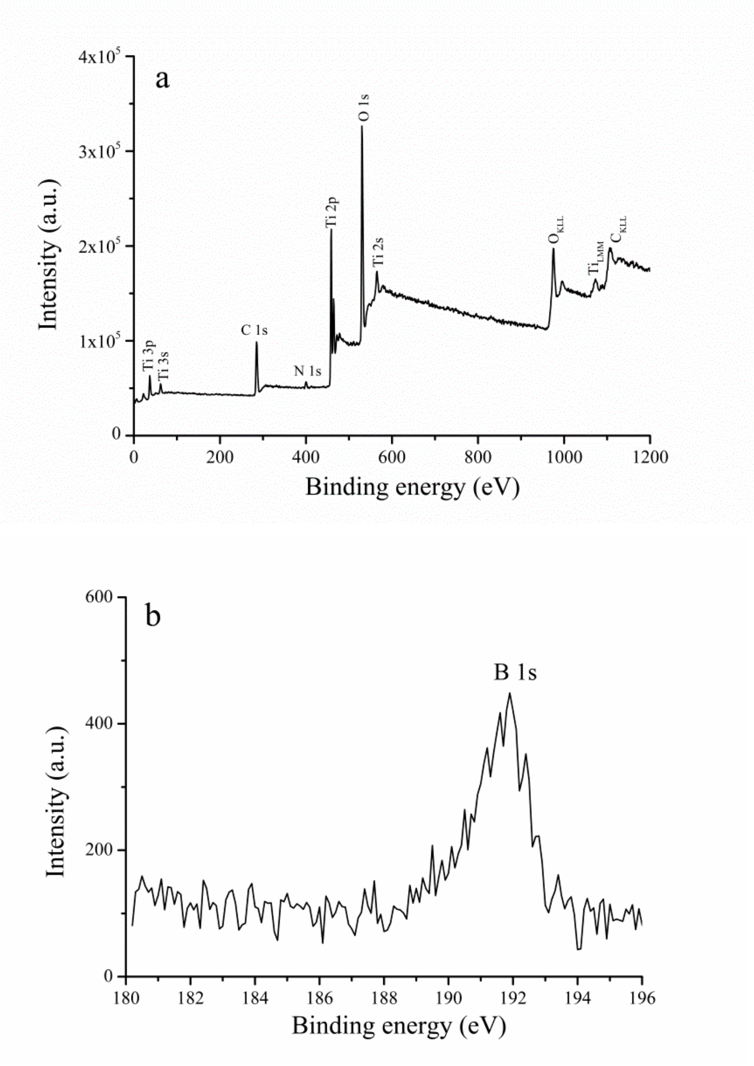

Figure S2. XPS survey spectrum (a) and high resolution spectrum of B 1s (b) of modified pollen grains.

Table S2 The amounts of enzymes immobilized on 1 mg of pollen grains (n=3).

	GOD adsorption (μg)	HRP adsorption (μg)
C_{GOD} in solution（0.7 μM）	15.82±1.20	
C_{HRP} in solution（0.7 μM）		6.12±0.24
C_{GOD} and C_{HRP} in solution（0.7 μM + 0.7 μM）	14.20±1.76	5.11±0.59

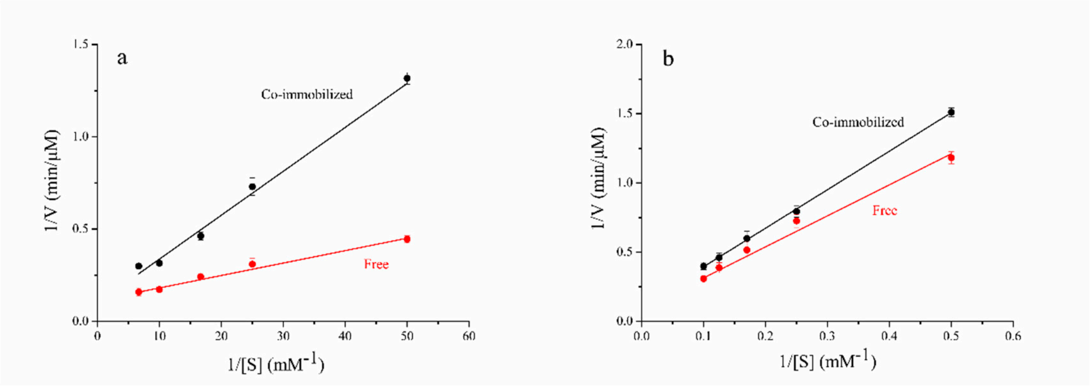

Figure S3. Enzymatic cascade reaction kinetics of free and immobilized GOD and HRP with (a) TMB as substrate and (b) glucose as substrate.

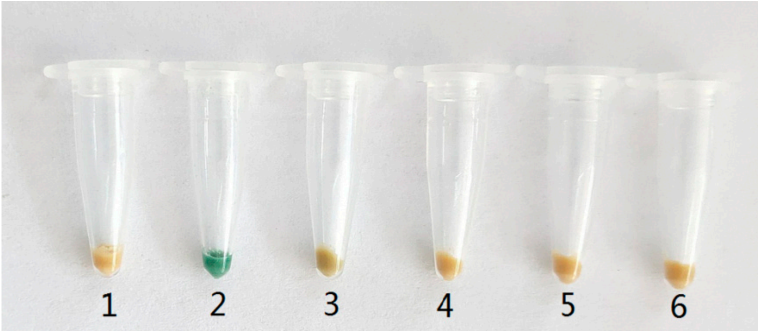

Figure S4. Digital image of the pollen grains in tubes for the detection of monosaccharides at different concentrations. (1) 0 mM glucose, (2) 30 mM glucose, (3) 0.3 mM glucose, (4) 30 mM mannose, (5) 30 mM sucrose, and (6) 30 mM ribose. For each of microcentrifuge tube, 20 μL of enzyme-immobilized pollen grain suspension, 20 μL of TMB solution (7.2 mM in deionized water), and 20 μL of glucose or other monosaccharide sample in 10 mM NH_4Cl buffer (pH 7.4) were added successively. Then the mixtures were incubated at 25°C for 6 min. After centrifugation at 6000 rpm for 1 min, the supernatants were drawn off.

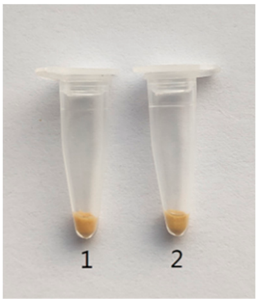

Figure S5. Digital image of modified pollen grains without enzyme (1) and HRP-immobilized pollen grains (2) in tubes for the detection of glucose. For each of microcentrifuge tube, 20 μL of pollen grain (without any enzyme) or only HRP-immobilized pollen grain suspension, 20 μL of TMB solution (7.2 mM in deionized water), and 20 μL of 30 mM glucose in 10 mM NH_4Cl buffer (pH 7.4) were added successively. Then the mixtures were incubated at 25°C for 6 min. After centrifugation at 6000 rpm for 1 min, the supernatants were drawn off.

Table S3 The recoveries of glucose in different samples (n=3).

Sample	Glucose added (mM)	Glucose found (mM)	R.S.D. (%)	LOD (mM)
Buffer	15	15.11±0.35	2.34	0.16
Urine	3	3.07±0.32	10.47	0.19

Table S4 The potential interfering substances in human body fluids [37].

Interferent	The normal range
D -Fructose	31 μM
Maltose monohydrate	1.2 mM
L -Cysteine	240–360 μM
Glutathione	0.065 μM
L -Phenylalanine	41.8–57.5 μM
Galactose	1.8 μM
Lactose	18 μM
Ascorbic acid	34 μM
Citric Acid	54 μM
Uric acid	0.5 mM
Glycine	0.49 μM
L -Tryptophan	7.8 μM
Potassium ion	3.5–5.0 mM
Sodium ion	135–150 mM
Magnesium ion	0.7–0.96 mM
Calcium ion	2.4–2.6 mM
L -Methionine	1 μM
L -Aspartic acid	10.9 μM
Lauric acid	0.03 μM
HSA	0.075 mM

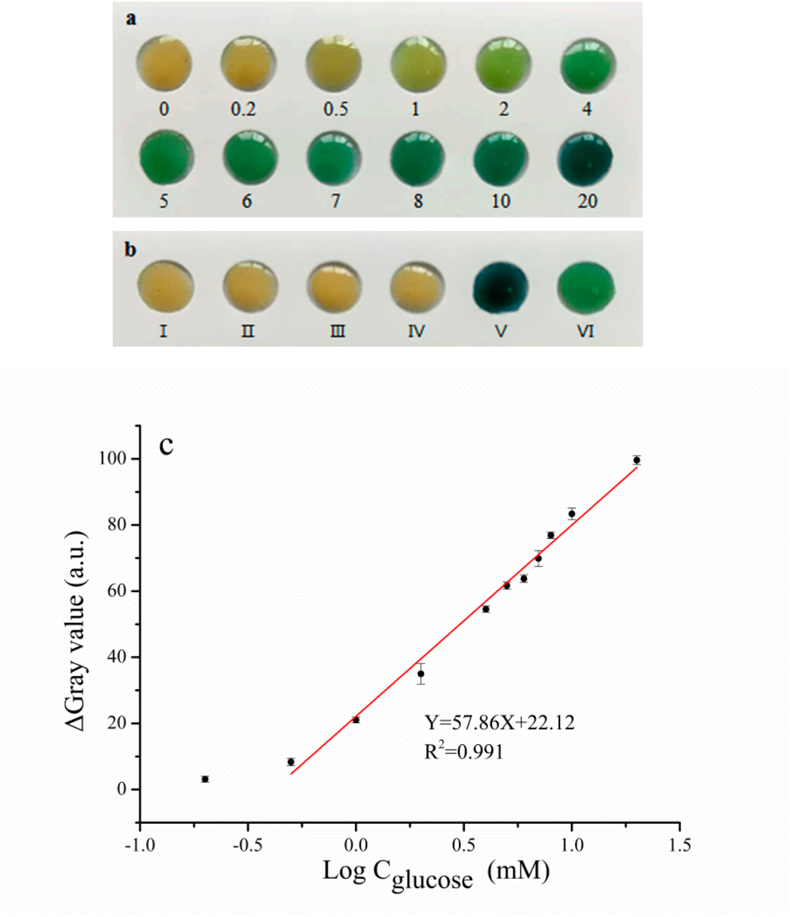

Figure S6. Digital images of the slides for (a) urine samples spiked with glucose and (b) urine samples from four healthy volunteers and two diabetics, and (c) the linearity of relative colour intensity with respect to glucose at different concentrations in urine samples. The concentration of glucose added in the urine sample was 0.2 to 20 mM, respectively. The error bars represent the mean ± SD of five independent experiments.

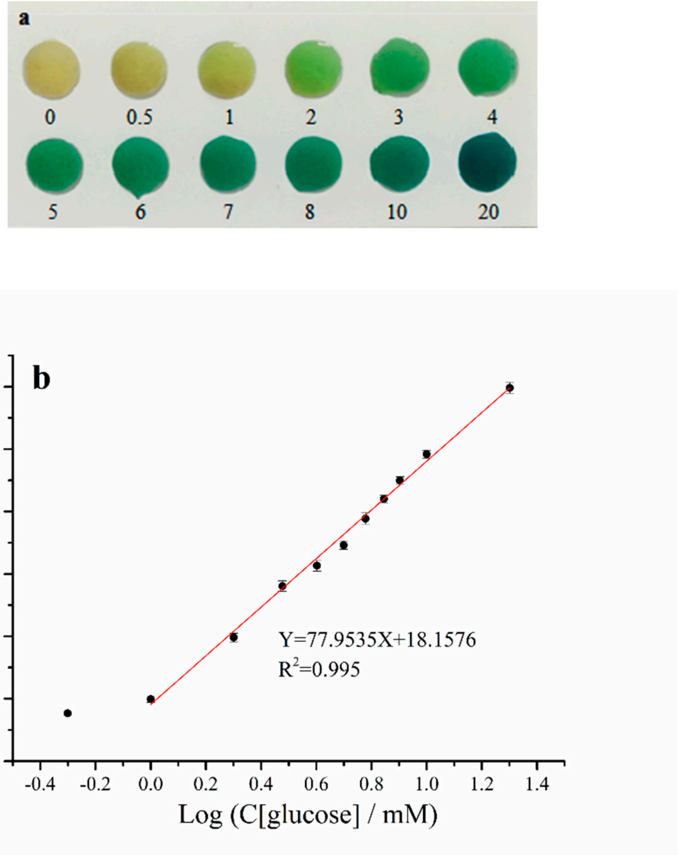

Figure S7. Digital image of the test slide for glucose at different concentrations spiked in diluted serum (a) and the linearity of relative color intensity with respect to glucose concentration (b). The concentration of glucose added in serum was 0.5 to 20 mM, respectively. The error bars represent the mean ± SD of five independent experiments.
